# Celastrol treatment protects against acute ischemic stroke-induced brain injury by promoting an IL-33/ST2 axis-mediated microglia/macrophage M2 polarization

**DOI:** 10.1186/s12974-018-1124-6

**Published:** 2018-03-14

**Authors:** Mei Jiang, Xinghui Liu, Denghai Zhang, Ying Wang, Xiaoxia Hu, Fengxia Xu, Mingming Jin, Fanfan Cao, Limin Xu

**Affiliations:** 1 Department of neurology, Shanghai Gongli Hospital, The Second Military Medical University, Shanghai, 200135 People’s Republic of China; 2 Department of Clinical Laboratory, Shanghai Gongli Hospital, The Second Military Medical University, 207 Ju Ye Road, Pudong New Area, Shanghai, 200135 People’s Republic of China; 3 Sino-French Cooperative Central Lab, Shanghai Gongli Hospital, The Second Military Medical University, 207 Ju Ye Road, Pudong New District, Shanghai, 200135 People’s Republic of China

**Keywords:** Celastrol, ST2, IL-33, M2 microglia/macrophage polarization, Acute ischemic stroke

## Abstract

**Background:**

Acute ischemic stroke (AIS) is the most common type of cerebrovascular disease and is a leading cause of disability and death worldwide. Recently, a study suggested that transformation of microglia from the pro-inflammatory M1 state to the anti-inflammatory and tissue-reparative M2 phenotype may be an effective therapeutic strategy for ischemic stroke. Celastrol, a traditional oriental medicine, may have anti-inflammatory and neuroprotective effects. However, the underlying mechanisms remain unknown.

**Methods:**

We first determined the expression levels of inflammatory factors in patients and rodent models associated with AIS; we then determined the anti-inflammatory effects of celastrol in AIS, both in vivo and in vitro, using animal models of middle cerebral artery occlusion (MCAO) and cell models of oxygen-glucose deprivation (OGD) treatment with or without celastrol, respectively.

**Results:**

The results indicated that expression of both inflammatory (interleukin (IL)-1β, IL-6, and tumor necrosis factor (TNF)-α) cytokines, as well as the anti-inflammatory cytokine, IL-33, and IL-10, were increased following AIS in patients and in animal models. Furthermore, in vitro experiments confirmed that celastrol treatment decreased inflammatory cytokine expression induced by OGD through an IL-33/ST2 axis-mediated M2 microglia/macrophage polarization. Finally, celastrol is protected against ischemic-induced nerve injury, both in vivo and in vitro.

**Conclusions:**

Taken together, these data suggest that celastrol post-treatment reduces ischemic stroke-induced brain damage, suggesting celastrol may represent a novel potent pharmacological therapy.

## Background

Stroke remains one of the leading causes of death and disability worldwide [1] with nearly 80% of cases characterized as ischemic. Cerebral ischemia results in an inflammatory response which plays an important role in subsequent neuronal death. Very early thrombolysis and mechanical thrombectomy have been shown to improve outcome after ischemic stroke; nevertheless, additional therapies are urgently needed, as most patients with ischemic stroke receive insufficient benefits from existing therapies [2]. Recently, post-ischemic inflammation, which lasts nearly a week after stroke onset, has been considered as a potential target in widening the therapeutic time frame [3, 4]. In addition, uncovering the mechanisms associated with this inflammatory response may provide preventative therapies against subsequent neuronal cell death in ischemia-related events, including AIS [5, 6].

Increasing evidence indicates that microglia and infiltrated macrophages play critical roles in regulating the immune and inflammatory responses after brain injuries [7]. Microglia/macrophages are known to have different phenotypes with distinct functions during the course of ischemic brain injury [8]. M2 microglia protect neighboring cells by removing cell debris and releasing trophic factors for brain repair, while chronically activated M1 microglia exacerbate brain injury by producing neurotoxic substances, although they participate in clearing cell debris at early stages after stroke [9]. Microglia/macrophage phenotype polarization is likely dependent on activation status, and balancing this polarization is a promising therapeutic strategy for stroke treatment.

Expression of interleukin (IL)-33, a member of the IL-1 cytokine family, is considered to be a warning sign [10], although the role of IL-33 in cerebral ischemic infarction remains controversial. A recent study found that IL-33 expression was increased in acute ischemic stroke (AIS) patients compared with healthy controls [11]. IL-33 expression in brain tissue also improved ischemic-induced brain injury by promoting M2 phenotype polarization and suppressing Th17 responses in mice [12]. Another recent study found that IL-33/growth stimulation expressed gene 2 (ST2) signaling activated beneficial M2 macrophage polarization after ischemia and subsequently reduced neuronal cell death. ST2 (growth stimulation expressed gene 2) is a member of the IL-1 receptor superfamily and is expressed on cardiomyocytes, as well as a large variety of immune cells, including M2 macrophages [13].

Celastrol is a quinone methide triterpene isolated from the root extracts of *Tripterygium wilfordii* (thunder god vine) and *Celastrus regelii* [14]. Increasing evidence suggests that celastrol possesses anti-inflammatory and anti-oxidant activities [15, 16]. Indeed, it was shown that celastrol had a protective effect against ischemic injury in a rat cerebral ischemia model [17].

This study aimed to determine whether celastrol has protective effects against AIS-induced injury. We assessed the expression of inflammatory factors in patients with AIS and also analyzed the effects of celastrol on microglial polarization and the inflammatory response, both in vitro and in a rat model of ischemic stroke. The results indicate that celastrol does have protective effects against AIS-induced injury and that these effects are related to an IL-33/ST2 axis-mediated microglia/macrophage M2 polarization.

## Methods

### Patients and clinical characteristics

Sixty first-ever AIS patients (34 females and 26 males; average age, 62.2 ± 8.2 years) were recruited from January 1, 2017, to September 31, 2017, at The Pudong New Area Gongli Hospital, Shanghai, China. All patients were diagnosed as having an acute cerebral infarction according to the World Health Organization criteria [18] and had symptoms within 72 h. Age- and sex-matched healthy individuals were also selected from the Pudong New Area Gongli Hospital (32 males and 28 females; average age, 58.4 ± 6.9 years). Body weight and height were recorded for subsequent calculation of body mass index (BMI) using a standard formula: BMI (kg/m^2^) = body weight (kg)/height (m^2^). Stroke severity was assessed using the National Institutes of Health Stroke Scale.

Blood samples (20 ml) were taken from the cubital vein within 24 h of symptom onset and used for further analysis. Whole blood was used for assessment of white blood cells (WBC), red blood cells (RBC), platelet (PLT) count, hemoglobin (Hb) concentration, and erythrocyte sedimentation rate (ESR).

Serum concentrations of total cholesterol (TC), triglycerides (TG), low-density lipoprotein cholesterol (LDL), and high-density lipoprotein cholesterol (HDL) were analyzed using Randox kits (Randox Laboratories Ltd., Crumlin, UK) with an automated RX Imola biochemical analyzer (Randox Laboratories, Ardmore, UK); and the resulting atherogenic index was calculated using a standard formula: AI = (TC-HDLC)/HDLC (AI, atherogenic index; TC, total cholesterol; HDLC, high-density lipoprotein cholesterol). The serum expression levels of the inflammatory factors, interleukin 33 (IL-33), interleukin 6 (IL-6), interleukin 1β (IL-1β), and tumor necrosis factor-α (TNF-α), interleukin 10 (IL-10), were measured with commercially available Enzyme-Linked Immuno Sorbent Assay (ELISA) kits (Sen-Xiong Company, Shanghai, China).

### Animals and ethics statement

Male Sprague–Dawley rats (230–280 g, 36 rats) were purchased from Shanghai Sippr Bk Laboratory Animals Co., Ltd., (Shanghai, China). All rats were allowed free access to food and water under controlled conditions (12/12 h light/dark cycle with humidity of 60 ± 5%, and a temperature of 22 ± 3 °C). All animals were treated in accordance with the Guide for the Care and Use of Laboratory Animals, and all experiments were approved and performed according to the guidelines of the Ethics Committee of Pudong New Area Gongli Hospital. All surgical procedures were performed under anesthesia, and every effort was made to minimize suffering. Rats were anesthetized by intraperitoneal injection of pentobarbital sodium (30 mg/kg).

### Murine models of middle cerebral artery occlusion

Animals were anesthetized by intraperitoneal injection of sodium pentobarbital. Body temperature was monitored and maintained at 36.5 °C to 37.5 °C. A modified model of middle cerebral artery occlusion (MCAO) was used to induce permanent focal ischemia, as previously described [19]. Briefly, the right middle cerebral artery (MCA) was occluded by inserting a monofilament nylon suture (diameter 0.24–0.26 mm) with a heat-rounded tip into the internal carotid artery, which was advanced further until it closed the origin of the MCA. Sham-operated rats underwent the same surgical procedure without insertion of the filament. No mortalities of rats were observed.

### Animal treatments

Cerebral ischemia and sham-operated animals were randomly assigned to either vehicle (12 rats) or celastrol groups (24 rats). Twelve rats from celastrol groups were administered with 1 mg/kg celastrol dissolved in 0.9% NaCl and 1% dimethylsulfoxide (DMSO) (InvivoGen, San Diego, CA, USA) immediately and 1 day post-operation through intraperitoneal injection. Rats were re-anesthetized and sacrificed 72 h (control: MCAO: MCAO+celastrol = 6: 6: 6) or 10 days (control: MCAO: MCAO+celastrol = 6: 6: 6) post-MCAO. Behavioral tests were performed 3, 5, 7, and 10 days after MCAO.

### Measurement of infarct volume

Measurements of infarct volumes were performed as previously described [13]. Briefly, infarct volume was determined with 2, 3, 5-triphenyltetrazolium chloride (TTC) 72 h post-MCAO (*n* = 6 in each group). Brain tissue was sliced into thick sections (1-mm-thick coronal sections) and stained with a 2% solution of TTC for 20 min at 37 °C, followed by fixation with 4% paraformaldehyde. TTC-stained sections were imaged and analyzed using Image Pro-Plus 5.1 analysis system (Media Cybernetics, NY, NY, USA). Lesion volumes were calculated using the following formula: [total infarct volume − (the volume of intact ipsilateral hemisphere–the volume of intact contralateral hemisphere)]/contralateral hemisphere volume × 100%.

### Behavioral tests

Sensorimotor functional recovery after stroke was measured before MCAO and also 3, 5, 7, and 10 days post-procedure. All behavioral tests were performed by an investigator blinded to the experimental groups. The rotarod (IITC Life Science, NY, NY, USA) test was performed to determine sensorimotor coordination. Briefly, rats were placed on an accelerated rotating rod with an increasing speed from 4 to 120 rpm within 5 min. Rats were tested three times daily with an intermission of 5 min, and latency to fall off the rotating rod was recorded. The data were expressed as mean values from three trials. The adhesive removal test was also employed [20]. In brief, a rat was placed in a cage for 1 min and an adhesive tape (50 mm^2^) was applied to the distal radial region of the right forelimb as a tactile stimulus. The time to contact and the time to remove the tape were both recorded. Each animal was tested three times with a cutoff time of 120 s per trial. The data are presented as the mean time to contact and the mean time to remove the tape on each testing day.

### Immunofluorescence

Cells or post-fixated brain slices with 4 μm thickness were incubated with ST2 (cat. nos. 6131; 1:200; mouse original), Iba-1 (cat. nos. 6756; 1:400), CD206 (cat. nos. 53206; 1:400), CD16 (cat. nos: 6641; 1:400), and NeuN (cat. nos. 6745; 1:400) antibodies (all from InvivoGen, San Diego, CA, USA) overnight at 4 °C, then incubated with conjugated secondary antibody for 1 h at room temperature in the dark. After several washes with phosphate-buffered saline (PBS), the slides were incubated with DAPI for 3 min and then mounted in glycerol. Slides were then visualized using fluorescence microscopy (excitation range from 490 to 495 nm and optimal emission range from 515 to 525 nm), and ten random fields in each section were analyzed. Then further statistical analysis in each independent experiment was performed.

### Primary neuron-microglia co-cultures and treatments

Primary neurons and microglia were prepared from the brains of mixed-sex embryonic day 17 Sprague–Dawley rats, as previously described [21]. The co-culture system was established by inserting a transwell chamber (Millipore, Bedford, MA, USA) with 3 μm pores into 12-well plates (Costar, NY, USA). Primary neurons were seeded (Costar) at a density of 1 × 10^3^ cells/well, and an equal amount of microglia were seeded in the transwell chamber. The chamber was inserted into the wells of the plate to allow communication between the two cell types. Co-culture systems were subjected to 3 h oxygen-glucose deprivation (OGD) or control treatment, after which 12.5, 25, 50, or 100 ng/mL IL-33 (Enzo Life Science, NY, USA) or PBS were added to transwell chambers for 24 h. In addition, celastrol (0.25, 0.5, 1, or 2 μM) or PBS were added 3 h after OGD or control treatment for 24 h to assess possible neuroprotective effects. Neurons were subsequently collected for cell viability assays.

### Primary microglia-enriched cultures and treatments

Primary microglia-enriched cultures were prepared from whole brains of 1-day old Sprague–Dawley rat pups as previously described [21]. Microglia were transfected with a ST2 interference vector before pretreatment with OGD for 3 h, then treated with 50 ng/mL IL-33, 1 μM celastrol, or PBS for 24 h. Cells were collected for microglial polarization analysis using immunofluorescent staining.

### Primary neuronal cultures and treatments

Primary neuronal cultures were prepared from 1-day old Sprague–Dawley rat pups as previously described [21]. Neurons underwent OGD for 3 h after which they were treated with different concentrations of IL-33 (12.5, 25, 50, or 100 ng/mL), celastrol (0.25, 0.5, 1, and 2 μM) or PBS for 24 h. Cell viability was assessed with the CCK8 assay.

### ELISA for soluble inflammatory cytokines

The expression of inflammatory factors IL-33, IL-6, IL-10, IL-1β, and TNF-α in cell supernatants or serum were measured using commercially available ELISA kits (Sen-Xiong Company). In accordance with the manufacturer’s instructions, supernatants were stored at − 80 °C before measurements and both standards and samples were run in triplicate. The optical density (OD)_450_ was calculated by subtracting the background, and standard curves were plotted.

### Cell viability assay

Cell counting kit-8 (CCK8) was used to assess cell viability. Cells (1 × 104) were seeded into 96-well plates, and grown overnight and treated as described previously in this section. Medium was removed, and the cells were washed three times with PBS. DMEM (90 μL) and CCK8 (10 μL) were subsequently added to each well and incubated for 1.5 h at 37 °C, and a microplate reader was used to measure the OD_450_.

### Apoptosis assay

Flow cytometry was used to determine the percentage of apoptotic cells. Apoptotic cells were differentiated from viable or necrotic cells by the combined application of annexin V (AV)-FITC (Roche, Indianapolis, IN, USA) and propidium iodide (PI) (Roche). Cells were washed twice and adjusted to a concentration of 1 × 10^6^ cells/mL with cold D-Hanks buffer. AV-FITC (10 μL) and PI (10 μL) were added to 100 μL of cell suspension and incubated for 15 min at room temperature in the dark. Finally, 400 μL of binding buffer was added to each sample without washing and analyzed with flow cytometry. Each experiment was performed at least in triplicate.

To detect apoptotic neurons, post-fixated brain slices (4 μm thickness) were measured with TUNEL detection kit (Roche) according to the manufacturer’s instructions. The nuclei were stained with DAPI, and TUNEL staining was then assessed. Nuclei that were double-labeled with DAPI and TUNEL were considered apoptotic.

### Western blotting analysis

Cells were harvested and lysed in Triton X-100 lysis buffer containing 25 mM Tris–HCl (pH 7.5), 137 mM NaCl, 2.7 mM KCl, 1% Triton X-100, and protease inhibitor cocktail (Sigma-Aldrich, St. Louis, MO, USA) for 30 min at 4 °C. Purified proteins were then detected by sodium dodecyl sulfate-polyacrylamide gel electrophoresis (SDS-PAGE) using a 12% separating gel and a 5% stacking gel followed by staining with Coomassie blue to determine the purity of the enzyme preparations. Western blotting analysis was performed as follows: the proteins were transferred from SDS-PAGE gels onto nitrocellulose membranes and blocked in TBS-T buffer (50 mM Tris–HCl pH 7.5, 150 mM NaCl and 0.2% Tween-20) for 1 h at room temperature with 5% nonfat milk. Membranes were then probed with primary antibodies, including ST2 and GAPDH, followed by incubation in respective secondary antibodies (Santa Cruz Biotechnologies Inc., Santa Cruz, CA, USA). Membranes were then incubated with Lumi-PhosTM WB (Pierce, Rockford, IL, USA) chemiluminescent substrate for 5 min. Protein expression levels were measured by densitometry using ImageJ 1.48u4 software (National Institutes of Health, NY, USA), and the data were normalized against the corresponding loading control (GAPDH).

### RNA extraction and qRT-PCR

Total RNA was extracted from cells using TRIzol reagent (Sigma-Aldrich) according to the manufacturer’s instructions. Total RNA was eluted with RNase-free water and stored at − 80 °C. RNA concentrations were determined with Epoch spectrophotometry. Quantification of ST2 and endogenous control mRNA U6 was performed using TaqMan assays with the supplied assay-specific RT primers (Applied Biosystems, Foster City, CA, USA). The data were analyzed using the 2^−ΔΔCt^ method.

### Construction and transfection of siRNAs

Specific siRNA against ST2—with the target sequence: 5′-CACTGGGAACTTATGTTGAATGG-3′—was synthesized by GenScript (Piscataway, NJ, USA). Microglia cells were dispersed into collagen I-coated six-well plates at a density of 5 × 10^4^ cells/well and cultured overnight under normal conditions. The following day, cells were transfected with siRNA in serum-free medium using oligofectamine reagent (Invitrogen) for 48 h at 37 °C with 5% CO_2_. Cells were then collected for further analysis.

### Statistical analysis

Data are presented as the mean ± standard deviation (SD). Statistical analysis was performed using one-way ANOVA with post hoc tests for comparisons between two groups using GraphPad Prism 5.0 software (GraphPad Software, Inc., La Jolla, CA, USA). *P* values ≤ 0.05 indicate statistically significant differences.

## Results

### Serum IL-33 levels and inflammatory factor levels are increased in stroke patients

Clinical test results showed that AIS did not alter blood Hb, RBC, or PLT counts, whereas a significant 70.5% increase in the WBC count was observed in patients suffering from AIS compared to the control values (Table 1). In addition, ESR in AIS exceeded control levels by a factor of more than three. No significant difference in serum TC, TG, HDL, or LDL levels were detected between the groups. ELISA results showed that serum levels of the anti-inflammatory cytokine, IL-33 (165 vs 283 pg/ml) and IL-10 (32.3 vs 68.4 pg/ml), as well as the inflammatory cytokines, IL-1β (23.5 vs 69.6 pg/ml), IL-6 (57.4 vs 122.8 pg/ml), and TNF-α (29.5 vs 82.5 pg/ml), were upregulated after AIS. According to previous reports, IL-33 might be involved in the inflammatory responses in acute cerebral infarction patients [11, 12].

**Table 1:** Clinicopathological characteristics of AIS and healthy controls

Characteristics	Controls (60)	AIS (60)
Age (years)	58.4 ± 6.9	62.2 ± 8.2
Sex		
Male	32	26
Female	28	34
Weight (kg)	78.9 ± 6.3	81.3 ± 7.8
Height (m)	1.7 ± 0.1	1.7 ± 0.1
BMI	26.8 ± 3.1	27.1 ± 3.4
NIHSS	–	12.6 ± 2.4
Risk factors (no)		
Hypertension, *n* (%)	–	46 (76.7%)
CHD, *n* (%)	–	22 (36.7%)
Laboratory findings		
WBC (10^9^/L)	6.1 ± 1.6	10.4 ± 7.2***
RBC (10^12^/L)	4.8 ± 0.3	4.9 ± 0.5
Hb (g/L)	139.3 ± 8.9	144.6 ± 11.8
PLT (10^9^/L)	236.3 ± 72.6	249.6 ± 68.6
ESR (mm/h)	5.6 ± 2.3	19.2 ± 8.8***
TC (mmol/L)	5.3 ± 0.4	4.9 ± 0.5
TG (mmol/L)	1.2 ± 0.12	1.4 ± 0.13
HDL (mmol/L)	1.0 ± 0.11	1.04 ± 0.08
LDL (mmol/L)	2.95 ± 0.82	2.97 ± 0.72
AI	2.7 ± 0.3	3.3 ± 0.4*
IL-33 (pg/mL)	165 ± 25.6	283 ± 54.8***
IL-1β (pg/mL)	23.5 ± 3.2	69.6 ± 3.4***
IL-6 (pg/mL)	57.4 ± 6.8	122.8 ± 11.6***
TNF-α (pg/mL)	29.5 ± 4.3	82.5 ± 7.9***
IL-10 (pg/mL)	32.3 ± 5.2	68.4 ± 6.2***

The data are presented as mean ± standard. *P < 0.05, ***P < 0.001 vs healthy controls

*AIS* acute ischemic stroke, *BMI* body mass index, *NIHSS* National Institutes of Health Stroke Scale, *CHD* coronary heart disease, *WBC* white blood cells, *RBC* red blood cells, *Hb* hemoglobin, *PLT* platelets, *ESR* erythrocyte sedimentation rate, *TC* total cholesterol, *TG* triglycerides, *HDL* high-density lipoproteins, *LDL* low-density lipoproteins, *AI* atherogenic index, *IL-33* interleukin-33, *IL-1β* interleukin-1β, *IL-6* interleukin-6, *TNF-α* tumor necrosis factor-α, *IL-10* interleukin-10

### Celastrol treatment prevents ischemic-induced brain injury in a rodent MCAO model

**Fig. 1:**
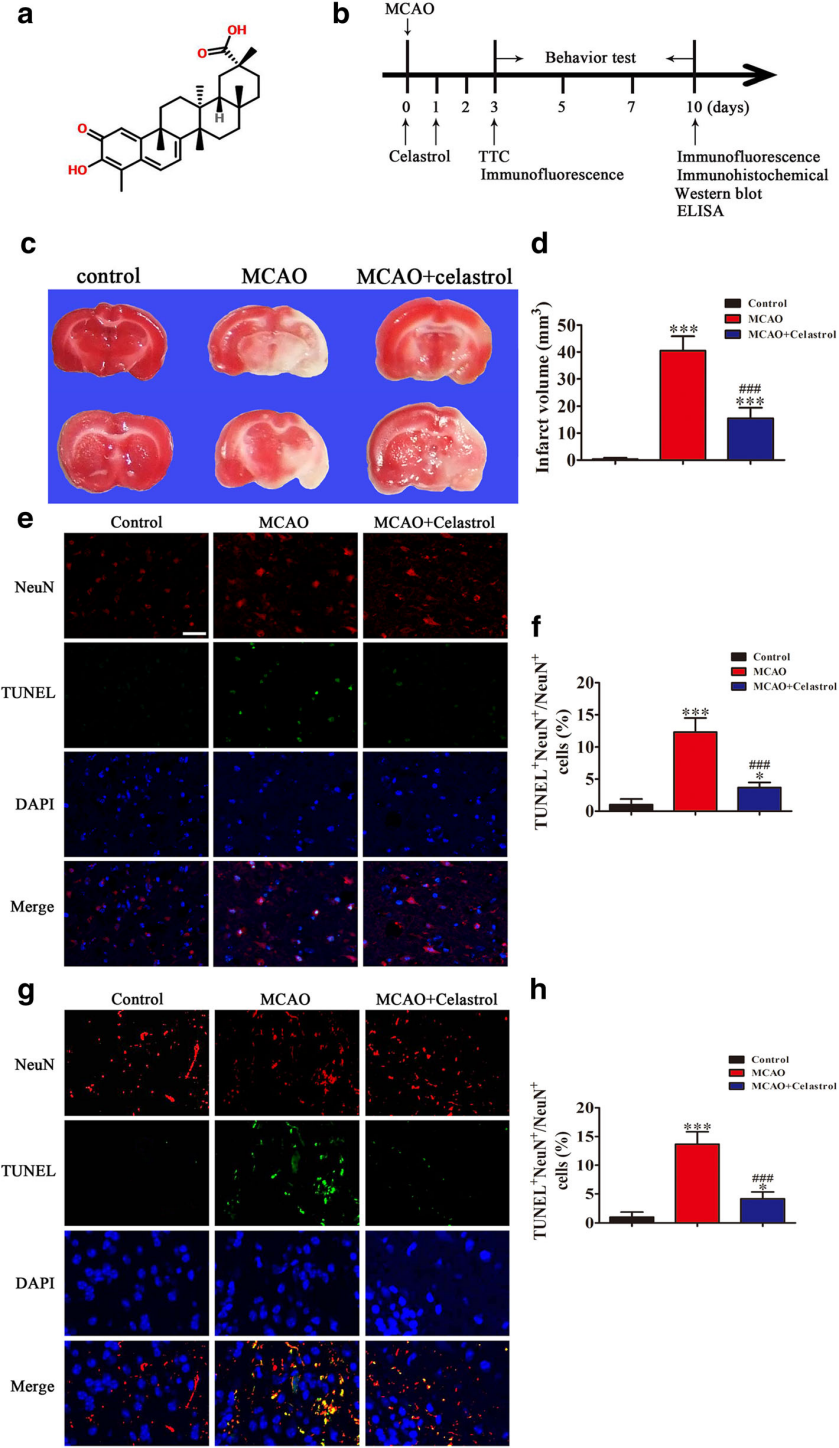
Celastrol treatment reduces infarct volume 3 days after middle cerebral artery occlusion (MCAO). **a** The structure of celastrol. **b** Experimental procedure for in vivo experiments. Stroke was induced in rats with permanent occlusion of the MCAO. Celastrol was intraperitoneally injected (1 mg/kg) at 0 and 24 h after ischemia. Three days after stroke, M1/M2 microglia markers and infarct volumes were measured with immunofluorescence and TTC staining, respectively. The adhesive removal test and modified Garcia scores were used to measure post-stroke sensorimotor functions 0, 3, 5, 7, and 10 days after cerebral ischemia. To confirm whether celastrol regulates microglial polarization in the brain after MCAO, brain sections were double-stained for Iba-1 (microglia) and CD206 (M2 marker) or CD16 (M1 marker) 10 days after stroke. ELISA analyses were used to detect the expression of serum IL-33 10 days after stroke. Immunohistochemistry was used to detect apoptotic neurons 10 days after stroke. **c** Representative TTC staining 3 days after MCAO. **d** Infarct volume in MCAO rats with or without celastrol. The data are presented as mean ± SD. ***P < 0.001 vs. control group. ^###^*P* < 0.001 vs. MCAO group. **e** Representative images of cortex around infarct regions with NeuN- and TUNEL-staining 3 days after MCAO treatment with or without celastrol. Scale bar = 50 μm. **f** The quantification of NeuN and TUNEL double-labeled cells. The data are presented as mean ± SD. *P < 0.05, ***P < 0.001 vs. control group. ^###^*P* < 0.001 vs. MCAO group. **g** Representative images of cortex around infarct regions with NeuN- and TUNEL-staining 10 days after MCAO treatment with or without celastrol. Scale bar = 50 μm. **h** The quantification of NeuN and TUNEL double-labeled cells. The data are presented as mean ± SD. *P < 0.05, ***P < 0.001 vs. control group. ^###^*P* < 0.001 vs. MCAO group. *n* = 6 rats

To identify the effects of celastrol in ischemic-induced brain injury, stroke was induced in rats by MCAO. Celastrol (Fig. 1a indicates the structure of celastrol) was injected at a concentration of 1 mg/kg at 0 and 24 h after ischemia, respectively. Three days after stroke, M1/M2 microglia markers and infarct volume were measured with immunofluorescence and TTC staining, respectively. Brain sections were double-stained for Iba-1 (microglia) and CD206 (M2 marker) or CD16 (M1 marker) 10 days after stroke. Immunohistochemical staining was used to detect neuronal apoptosis 10 days after stroke (Fig. 1b). The results show that infarct volumes were significantly larger in the MCAO group compared with controls, but celastrol treatment significantly decreased MCAO-induced infarct volume from 40 to 20% (Fig. 1c, d). The results from the TUNEL assay further confirmed that celastrol treatment prevented ischemia-induced neuronal apoptosis (Fig. 1e–h).

### Celastrol treatment protects against cerebral ischemia-induced neurological dysfunction

**Fig. 2:**
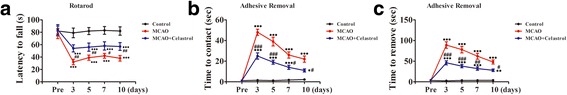
Celastrol treatment significantly improves sensorimotor functions early after MCAO. **a** Rotarod test. The data are presented as mean ± SD. ***P < 0.001 vs control group. ^#^*P* < 0.05, ^##^*P* < 0.01 vs. MCAO group. **b** and **c** Adhesive removal test. The data are expressed as the latency to contact (**b**) and removal (**c**) tape from the impaired forepaw. The data are presented as mean ± SD. *P < 0.05, ***P < 0.001 vs control group. ^#^*P* < 0.05, ^##^*P* < 0.01, ^###^*P* < 0.001 vs. MCAO group. *n* = 6 rats

Sensorimotor tests, including the rotarod (Fig. 2a) and adhesive removal (Fig. 2b, c), consistently revealed impaired neurological performance up to 10 days after stroke, but celastrol treatment improved ischemia-induced sensorimotor dysfunctions. The control group exhibited no significant differences in any behavioral tests during the different times tested, suggesting equivalent baselines in both groups.

### Celastrol treatment promotes ST2/IL-33 activation in microglia and promotes M2 microglial polarization

**Fig. 3:**
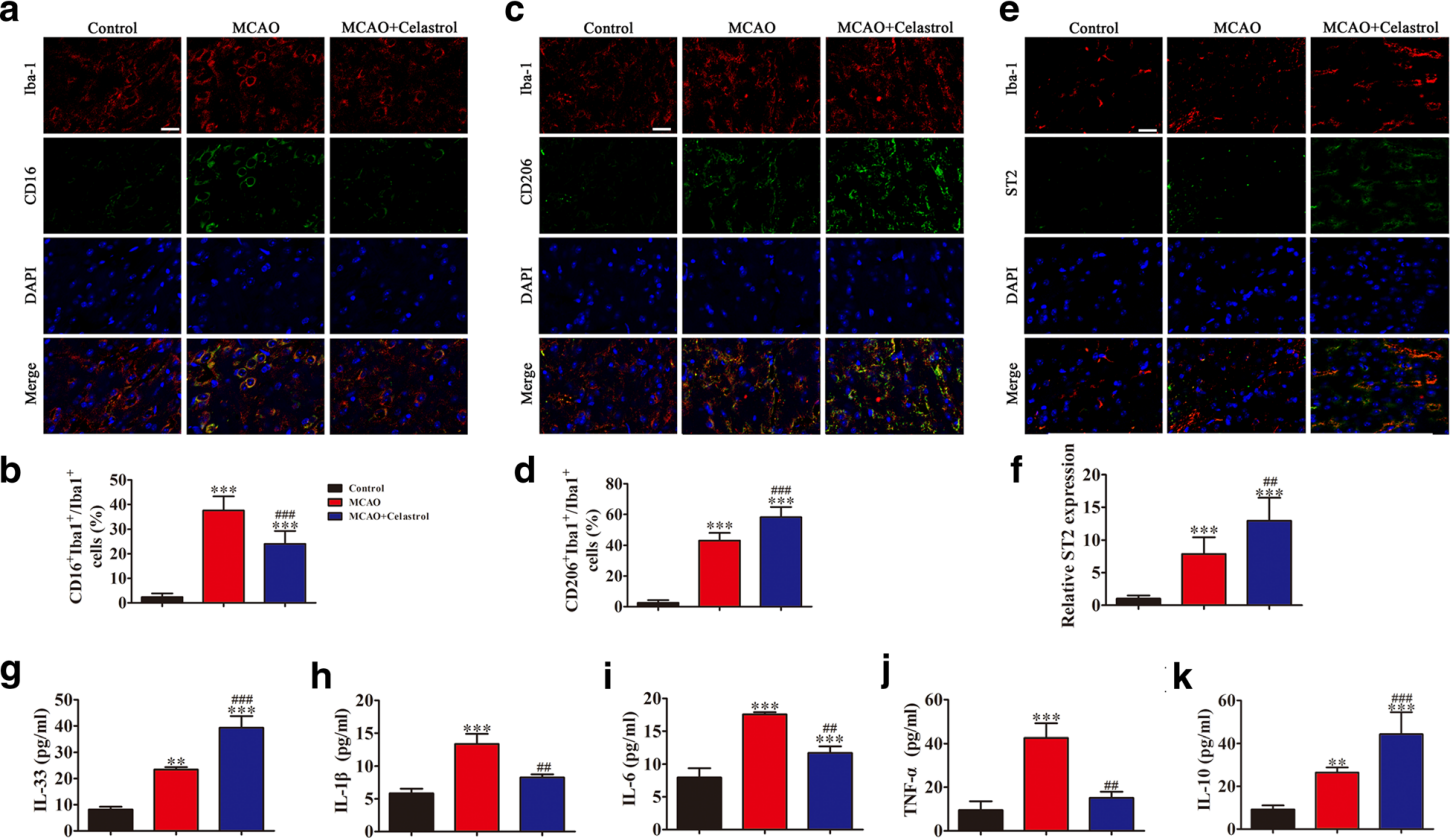
Celastrol treatment significantly promotes ST2/IL-33 activation in microglia and promotes microglial M2 polarization 10 days after stroke. Microglia were double-stained with CD16 or CD206 and Iba-1 in brain sections obtained from celastrol or vehicle-treated rat 10 days after MCAO, or from sham-operated rat. **a** Representative cortical sections double-stained with CD16 (M1 marker) (green) and Iba-1(red). Scale bar = 20 μm. **b** Quantification of the percentage of CD16^+^/Iba-1^+^ cells among total Iba-1^+^ cells. The data are presented as mean ± SD. ^***^P < 0.001 vs. control group. ^###^*P* < 0.001 vs. MCAO group. **c** Cortical sections double-stained with CD206 (M2 marker) (green) and Iba-1 (red). Scale bar = 20 μm. **d** Quantification of the percentage of CD206^+^/Iba-1^+^ cells among total Iba-1^+^ cells. The data are presented as mean ± SD. ^***^P < 0.001 vs. control group. ^###^*P* < 0.001 vs. MCAO group. **e** Cortical sections double-stained with ST2 (green) and Iba-1 (red). Scale bar = 20 μm. **f** Quantification of mean fluorescence intensity of ST2-positive microglia. The data are presented as mean ± SD. ^***^P < 0.001 vs. control group. ^##^*P* < 0.01 vs. MCAO group. (G-K) ELISA results showing the expression of serum IL-33 (**g**), IL-1β (**h**), IL-6 (**i**), TNF-α (**j**), and IL-10 (**k**). The data are presented as mean ± SD. ^**^P < 0.01, ^***^P < 0.001 vs. control group. ^##^*P* < 0.01, ^###^*P* < 0.001 vs. MCAO group. *n* = 6 rats

In order to determine whether the protective effect of celastrol was related to a ST2/IL-33 axis-mediated microglial phenotypic shift (as previously reported) [13], we used immunofluorescence to detect microglial phenotypic markers in brain sections of rat sacrificed 10 days after MCAO. Results indicate that celastrol treatment reduced expression of the M1 marker, CD16, (Fig. 3a, b), but increased expression of the M2 marker, CD206 (Fig. 3c, d). The data also show that celastrol treatment increased ST2 expression in microglia (Fig. 3e, f). ELISA detection showed that comparing with MCAO group, celastrol treatment increased IL-33 (8.2 pg/ml (control): 23.4 pg/ml (MCAO): 39.3 pg/ml (MCAO+Celastrol)) and IL-10 (9.2 pg/ml (control): 26.4 pg/ml (MCAO): 44.2 pg/ml (MCAO+Celastrol)) expression in serum levels (Fig. 3g, k), but decreased IL-1β (5.9 pg/ml (control): 13.4 pg/ml (MCAO): 8.3 pg/ml (MCAO+Celastrol)) (Fig. 3h), IL-6 (8.0 pg/ml (control): 17.6 pg/ml (MCAO): 11.7 pg/ml (MCAO+Celastrol)) (Fig. 3i), and TNF-α (9.5 pg/ml (control): 42.6 pg/ml (MCAO): 15.1 pg/ml (MCAO+Celastrol)) (Fig. 3j) levels in serum.

### ST2 signaling in microglia is essential for the M2 phenotype in microglia/macrophages

**Fig. 4:**
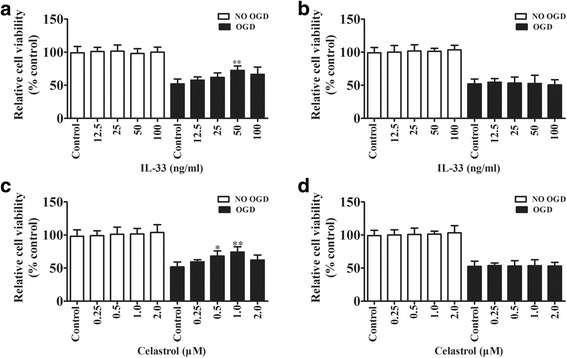
IL-33/ST2 signaling in microglia is essential for celastrol treatment-induced neuroprotective effects in vitro. Neuron-microglia co-cultures or neuron only cultures with or without OGD for 3 h were treated with a range of IL-33 concentrations, celastrol, or PBS (control), respectively, for 24 h. **a** Neuronal activity in IL-33-treated neuron-microglia co-cultures was detected using CCK8. The data are presented as mean ± SD. ^**^P < 0.01 vs. control group. **b** Neuronal activity in IL-33-treated neuron exposed to OGD for 3 h was detected using CCK8. **c** Neuronal activity in celastrol-treated neuron-microglia co-cultures was detected using CCK8. The data are presented as mean ± SD. ^*^P < 0.05, ^**^P < 0.01 vs. control group. **d** Neuronal activity in celastrol-treated neuron exposed to OGD for 3 h was detected using CCK8. Each experiment repeated 6 times

Results from neuron/microglial co-cultures and neuron cultures with or without OGD conditions for 3 h showed that both IL-33 (Fig. 4a) and celastrol (Fig. 4c) treatment at various concentrations are protected against OGD-induced neuronal cell death in neuron-microglia co-cultures, although no effects were found in neuron only cultures (IL-33 treatment (Fig. 4b); celastrol treatment (Fig. 4d)). These results also suggested that the optimal concentration of IL-33 and celastrol were 50 mg/mL and 1 μM, respectively.

**Fig. 5:**
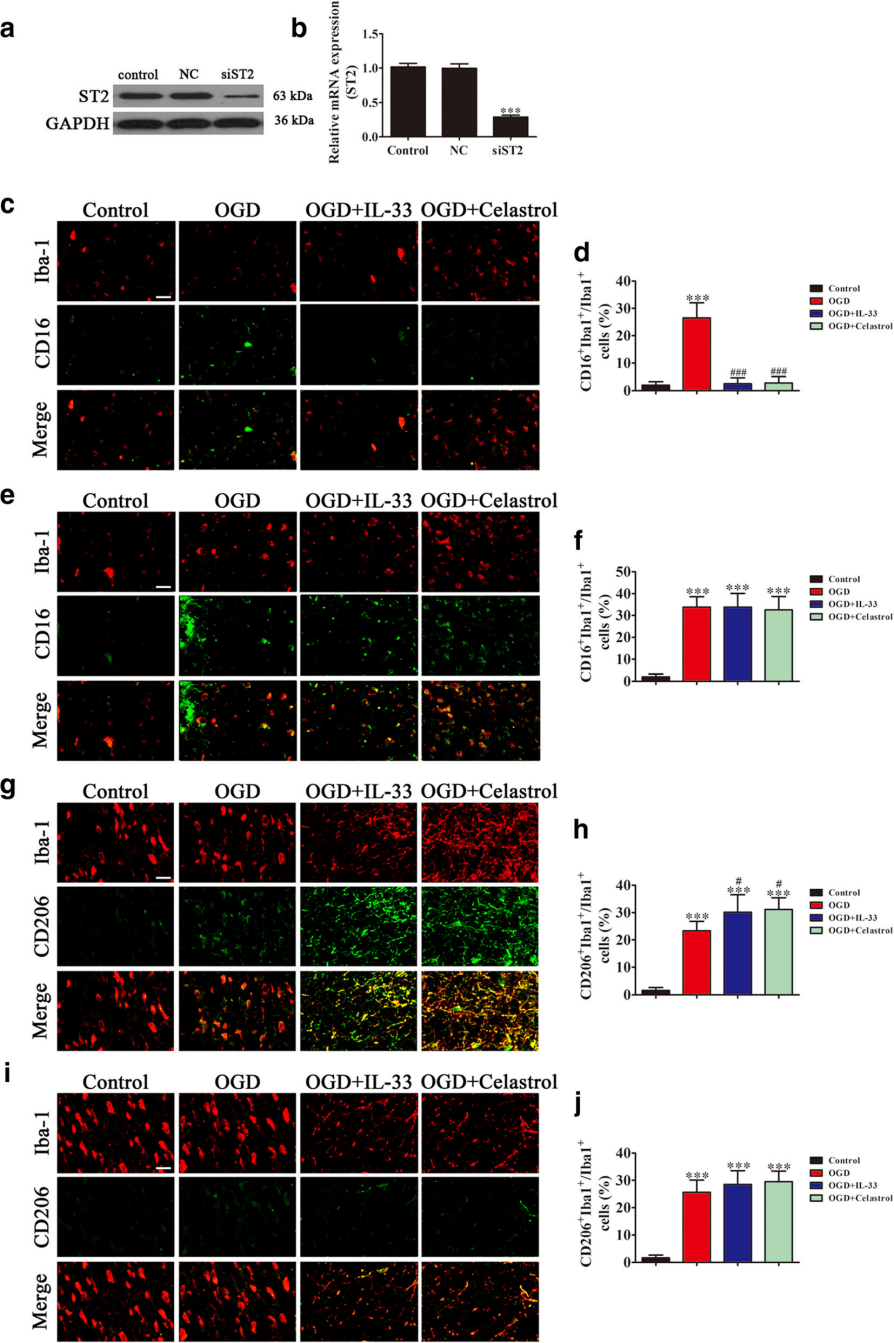
ST2 signaling is essential for M2 polarization after ischemia. Microglial cells transfected with or without siRNA against ST2 were exposed to OGD conditions for 3 h before treatment with either IL-33 (50 ng/mL), celastrol (1 μM), or PBS (control) for 24 h. Western blot (**a**) and qRT-PCR (**b**) were used to detect the expression of ST2 after transfection with siRNA against ST2. The data are presented as mean ± SD. ^***^P < 0.001 vs. control group. **c** Representative images of double immunofluorescent staining of microglia with the microglia/macrophage marker, Iba1 (red), and the M1 marker, CD16 (green). Scale bar = 50 μm. **d** Quantification of the percentage of CD16^+^/Iba-1^+^ cells among total Iba-1^+^ cells. The data are presented as mean ± SD. ^***^P < 0.001 vs. control group. ^###^*P* < 0.001 vs. OGD group. **e** Representative images of double immunofluorescent staining of microglia with the microglia/macrophage marker, Iba1 (red), and the M1 marker, CD16 (green), after transfection with siRNA against ST2. Scale bar = 50 μm. **f** Quantification of the percentage of CD16^+^/Iba-1^+^ cells among total Iba-1^+^ cells. The data are presented as mean ± SD. ^***^P < 0.001 vs. control group. **g** Representative images of double immunofluorescent staining of microglia with the microglia/macrophage marker, Iba1, and the M2 marker, CD206. Scale bar = 50 μm. **h** Quantification of the percentage of CD206^+^/Iba-1^+^ cells among total Iba-1^+^ cells. The data are presented as mean ± SD. ^***^P < 0.001 vs. control group. ^#^*P* < 0.05 vs. OGD group. **i** Representative images of double immunofluorescent staining of the microglia/macrophage marker, Iba1, and the M2 marker, CD206, after transfection with siRNA against ST2. Scale bar = 50 μm. **j** Quantification of the percentage of CD206^+^/Iba-1^+^ cells among total Iba-1^+^ cells. The data are presented as mean ± SD. ^***^P < 0.001 vs. control group. Each experiment repeated 6 times

To further explore the effects of ST2 on microglia/macrophage phenotypic responses after stroke, siRNAs against ST2 were transfected into microglia before OGD exposure and treatment with IL-33 (50 ng/mL) or celastrol (1 μM), respectively. Transfection with siRNA significantly decreased both ST2 protein (Fig. 5a) and mRNA levels (Fig. 5b). Immunofluorescent detection showed that celastrol promoted the M2 phenotype under OGD conditions at the expense of the M1 phenotype (Fig. 5c–f). Downregulation of ST2 promoted the M1 phenotype under OGD conditions even after treatment with IL-33 or celastrol (Fig. 5g–j).

### Celastrol treatment protects against OGD-induced neuronal apoptosis and inflammatory factor expression

**Fig. 6:**
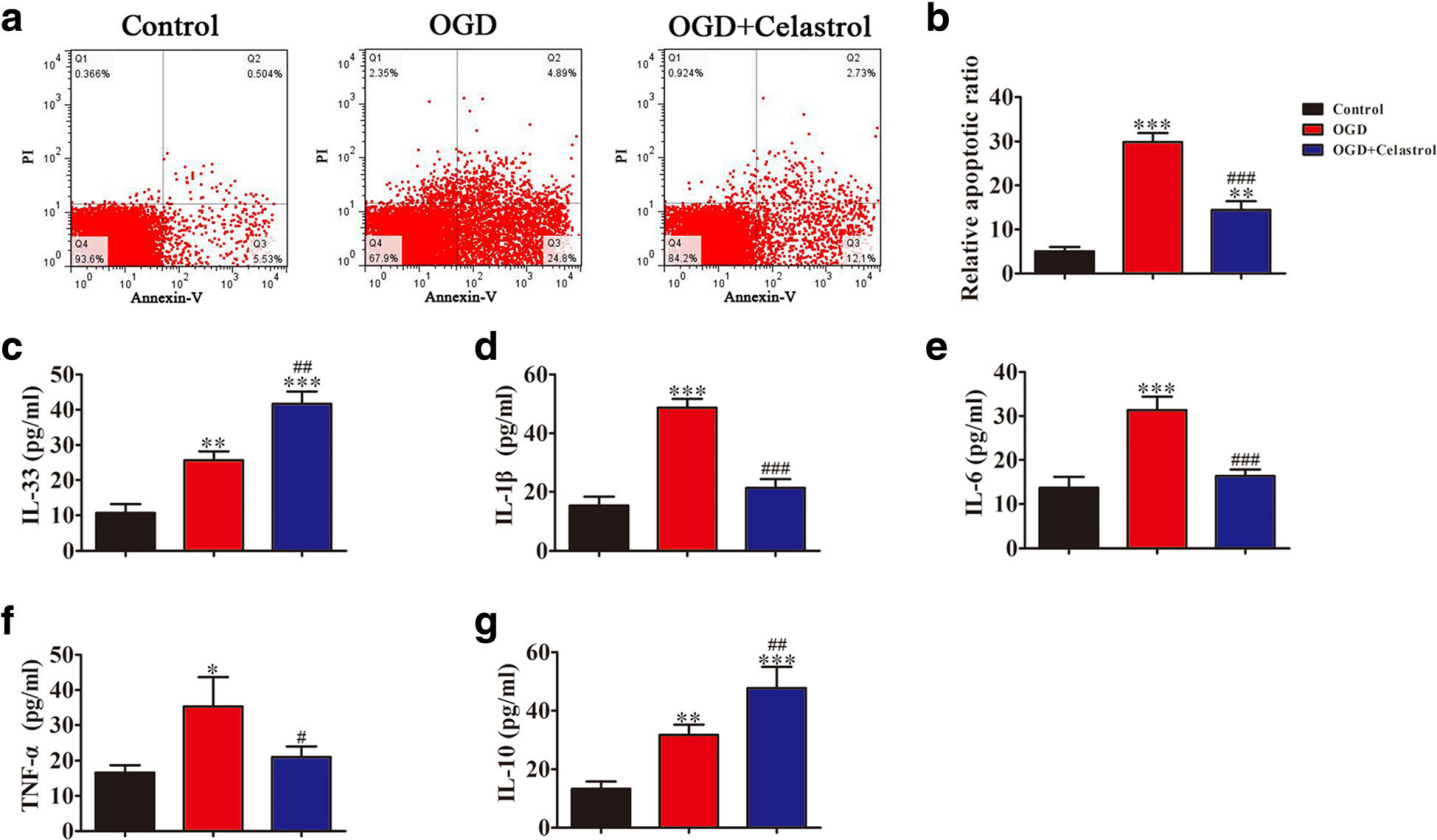
Celastrol treatment protects against OGD-induced neuronal apoptosis and inflammatory factor expression. Neuron-microglia co-cultures exposed to OGD conditions for 3 h were treated with celastrol (1 μM) or PBS (control) for 24 h. **a** Neuronal apoptosis was assessed with flow cytometry using annexin V-FITC staining, and **b** the relative apoptosis ratio was analyzed. The data are presented as mean ± SD. ^**^P < 0.01, ^***^P < 0.001 vs. control group. ^###^*P* < 0.001 vs. OGD group. **c**–**g** ELISA results showing the expression of IL-33 (**c**), IL-1β (**d**), IL-6 (**e**), TNF-α (**f**), and IL-10 (**g**) in cell supernatant. The data are presented as mean ± SD. ^*^P < 0.05, ^**^P < 0.01, ^***^P < 0.001 vs. control group. ^#^*P* < 0.05, ^##^*P* < 0.01, ^###^*P* < 0.001 vs. OGD group. Each experiment repeated 6 times

To confirm whether celastrol treatment suppressed ischemia-induced neuronal damage and inflammatory factor expression, neuron-microglial co-cultures exposed to OGD conditions for 3 h were treated with celastrol (1 μM) for 24 h. Results showed that celastrol treatment significantly suppressed neuronal apoptosis under OGD conditions (Fig. 6a, b). ELISA detection showed that the expression of IL-33 (10.1 pg/ml (control): 25.7 pg/ml (OGD): 41.7 pg/ml (OGD + Celastrol)) and IL-10 (13.3 pg/ml (control): 31.6 pg/ml (OGD): 47.7 pg/ml (OGD + Celastrol)) were increased (Fig. 6c, f), while expression of IL-1β (15.3 pg/ml (control): 48.7 pg/ml (OGD): 21.3 pg/ml (OGD + Celastrol)) (Fig. 6d), IL-6 (13.7 pg/ml (control): 31.3 pg/ml (OGD): 16.3 pg/ml (OGD + Celastrol)) (Fig. 6e), and TNF-α (16.7 pg/ml (control): 35.3 pg/ml (OGD): 21.0 pg/ml (OGD + Celastrol)) (Fig. 6f) decreased after celastrol treatment.

Taken together, these in vitro studies strongly suggest that activation of the IL-33/ST2 axis in microglia promotes polarization toward the M2 phenotype and protects neighboring neurons from ischemic injury.

## Discussion

Celastrol is a natural compound whose therapeutic potential has been reported in many diseases, including cancer, diabetes, and neurodegenerative disorders [16, 22, 23]. A growing number of reports suggest that celastrol protects against stroke-induced brain damage [17], and in agreement, here, we report that celastrol treatment not only ameliorated infarct volumes but also decreased the inflammatory response after ischemic stroke.

It is well known that inflammation response play key roles in ischemia-induced nerve injury. A series of studies were conducted to investigate anti-inflammatory effects of a therapy against ischemic damage [24, 25]. Previous research showed that microglia are divided into activated M1 and M2 microglia, according to their phenotypes and polarization [26]. Specifically, activated M1 microglia secreted pro-inflammatory cytokines and were potentially harmful, whereas activated M2 microglia served important roles in repair and plasticity [8, 27].

Recent studies have shown that IL-33 is released from CNS cells rapidly after injury and contributes to the activation of immune responses in lesion areas [28]. Previous work showed that IL-33/ST2 signal activation promoted microglial polarization toward the M2 phenotype, while at the same time, actively inhibited the expression of M1-mediated cytokines [13]. Interestingly, our result shows that IL-33 is increased along with other pro-inflammatory cytokines in serum of patients after stroke. Similarly, rat IL-33 axis is also increased after ischemia, and a further increase induced by celastrol induces M2 microglia phenotype. The anti-inflammatory effects of celastrol on microglial polarization and IL-33/ST2 signal activation remain unclear. Nevertheless, we showed IL-33 promoted microglial ST2 expression, which suppressed neuronal damage by promoting anti-inflammatory cytokine IL-10 expression after induced microglial polarization toward the M2 phenotype. Downregulation of ST2 promoted microglial M1 polarization even when high levels of IL-33 were present. Finally, celastrol treatment increased IL-33 expression and suppressed ischemia-induced inflammatory factor expression by promoting IL-33/ST2-mediated M2 microglial polarization. Therefore, we speculate that the protective effects of celastrol in AIS-induced brain injury are related to an IL-33/ST2 axis-mediated microglia/macrophage M2 polarization. Previous studies have implicated IL-33/ST2 axis in downregulation of TLR signaling [29]. The activation of TLR signaling can shift macrophage polarization toward the M1 phenotype [30]. But if TLR signaling is involved in IL-33/ST2 axis-mediated macrophage polarization, regulation is still unclear. So, in future work, we will generate a *ST2* knockout rat model to confirm the above finding and further elucidate the related mechanism of IL-33/ST2-mediated M2 microglial polarization.

## Conclusion

In conclusion, we found an increase in serum IL-33 and inflammatory factor levels in AIS patients. Celastrol treatment suppressed AIS-induced pathological nerve injury and sensorimotor dysfunctions by inhibiting inflammatory factor expression. In addition, celastrol markedly reduced ischemia-induced neuronal apoptosis and inflammatory factor expression, which was relative to IL-33/ST2-mediated M2 polarization. Taken together, these data show that celastrol is a promising new agent for preventing AIS-induced brain injury.

## Abbreviations

AIS: Acute ischemic stroke
CCK8: Cell counting kit-8
ELISA: Enzyme-Linked Immuno Sorbent Assay
ESR: Erythrocyte sedimentation rate
Hb: Hemoglobin
HDL: High-density lipoprotein cholesterol
IL-10: Interleukin-10
IL-1β: Interleukin-1β
IL-33: Interleukin-33
IL-6: Interleukin-6
LDL: Low-density lipoprotein cholesterol
MCAO: Middle cerebral artery occlusion
OGD: Oxygen-glucose deprivation
PBS: Phosphate-buffered saline
PLT: Platelet
RBC: Red blood cells
SDS-PAGE: Sodium dodecyl sulfate-polyacrylamide gel electrophoresis
TC: Total cholesterol
TG: Triglycerides
TNF-α: Tumor necrosis factor-α
TTC: 2, 3, 5-Triphenyltetrazolium chloride
WBC: White blood cells
